# The Association of New Onset Postoperative Atrial Fibrillation and Abnormal P-Terminal Force in Lead V1 After On-Pump Cardiac Surgery

**DOI:** 10.1177/10892532251321062

**Published:** 2025-02-14

**Authors:** Mirjana Gander, Joanna Kochanska-Bieri, Firmin Kamber, Denis Berdajs, David Santer, Daniel Bolliger, Eckhard Mauermann

**Affiliations:** 1 Medical School, 89389 University of Basel, Basel, Switzerland; 2 Clinic for Anesthesia, Intermediate Care, Prehospital Emergency Medicine and Pain Therapy, 30262 University Hospital Basel, Basel, Switzerland; 3 Clinic for Cardiac Surgery, 30262 University Hospital Basel, Basel, Switzerland; 4 Institute of Anesthesiology, Zurich City Hospital, Zurich, Switzerland

**Keywords:** cardiac surgery, postoperative atrial fibrillation, p-terminal force vector in lead V1, cardiopulmonary bypass, electrocardiography

## Abstract

**Introduction:** Postoperative atrial fibrillation (POAF) after cardiac surgery is associated with higher morbidity and mortality. This paper presents several studies that conclude the presence of an aberrant p-terminal force vector in lead V1 (PTFV1) has been identified as a significant predictor of atrial fibrillation in the non-surgical population. It is uncertain whether or not there is an association of PTFV1 and new-onset POAF in patients after cardiac surgery. **Methods:** In this secondary analysis, adult patients undergoing on-pump cardiac surgery for aortocoronary bypasses, valve surgery, combined bypass, and valve surgery were analyzed from 12/2018 to 08/2020. Patients who had a previous occurrence of atrial fibrillation or atrial flutter, patients with pacemakers and/or Implantable Cardioverter-Defibrillators (ICDs), and those who did not have an electrocardiogram (ECG) performed within the 3 months before surgery were excluded. In addition, ECGs that were considered to be of low quality were also removed. Preoperative 12-lead ECGs were examined and the PTFV1 was measured. Secondarily, we examined the P-wave length in lead II, the area under the P-wave in lead II, PR interval, and QRS duration in lead V1 and II. The occurrence of POAF was extracted from the hospital record. **Results:** Out of a total of 252 patients, 62 patients (24.6%) developed new onset POAF during their hospital stay. POAF occurred primarily in older patients, with poor renal function, and exhibited larger left atria. Analysis of ORs (odds ratios) revealed that age, creatinine clearance, valve surgery, and left atrial volume index (LAVI) were associated with POAF. In the context of the multivariable analysis, it was demonstrated that only age presented a significant correlation with postoperative atrial fibrillation (POAF). There was no observed relationship between any of the parameters based on ECG and the occurrence of POAF. **Conclusion:** No association was found between PTFV1 or other ECG-based measurements and new onset POAF in cardiac surgery patients. Age was the only independent predictor of POAF.

## Introduction

New-onset POAF after cardiac surgery is arguably the most common adverse event, with up to 30 to 50% of patients developing POAF within the first postoperative week.^1,2^ POAF is not only associated with both longer length of Intensive Care Unit (ICU) and hospital stay^3,4^ but also higher morbidity and mortality.^5,6^ As a result, postoperative atrial fibrillation has been a focus of international and multidisciplinary guidelines.^7,8^

Some predictors for the occurrence of POAF exist and have been moderately useful in defining elevated risk. These include the well-known CHADS and CHA_2_DS_2_-VASC scores as well as the POAF Score, which was specifically derived for this purpose.^9,10^ The CHA2DS2-VASC-Score is a tool used to assess the clinical risk for a thromboembolism in patients having atrial fibrillation.^11^ The POAF Score sums points (Age 60–69: 1 pt.; Age 70–79: 2 pts; Age >=80: 3 pts; Chronic Obstructive Pulmonary Disease [COPD]: 1 pt, estimated Glomerular Filtration Rate [GFR]<15 mL/min: 1 pt.; emergency: 1pt.; preop intra-aortic Balloon Pump (IABP) 1pt.; Left Ventricular Ejection Fraction [LVEF] <30: 1pt.; Valve surgery: 1 pt; >=3pts with POAF in >40%).

In addition to scores based upon a patient’s characteristics, history, and medical procedures, morphological features of the heart (e.g., atrial strain in echo and Magnetic Resonance Imaging [MRI])^12^ and the heart’s electrical activity (e.g., ECG)^13^ have in part shown incremental value in predicting POAF. While some imaging modalities may not be available or require expensive and time-consuming software, the preoperative ECG is routinely available, requires no specialized hardware/software, and is both inexpensive and non-invasive. Indeed, many ECG P-wave measurements have been associated with higher rates of atrial fibrillation in non-surgical patients.^14^ Recently, the abnormal PTFV1 has emerged in the non-surgical population as a predictor of atrial fibrillation in a large meta-analysis.^15^ However, the utility of the PTFV1 for predicting new onset POAF in patients undergoing cardiac surgery has not been examined.

We hypothesized that an abnormal PTFV1 would be associated with a higher risk of POAF in patients undergoing on-pump cardiac surgery. In this study, we aimed to (1) examine the association of an abnormal PTFV1 with POAF in the surgical population, (2) compare other ECG-based predictors with POAF, and (3) examine a possible incremental value with other POAF predictors all in a cardiac surgery population.

## Materials and Methods

### Study Design and Participants

This is a secondary analysis of an observational single-center, retrospective study approved by the Ethics Committee of Northwestern and Central Switzerland (EKNZ) with an informed consent waiver (EKNZ, 2016/1550, January 10, 2017).^16^ This original study included adult patients undergoing elective and non-elective on-pump cardiac surgery with median sternotomy at the University Hospital Basel, Switzerland, from December 2018 to August 2020. Aortocoronary bypasses, valve surgery, combined bypass, and valve surgery were included in the original study; patients with thoracotomies, partial sternotomies, and aortic surgery were excluded.

In this secondary analysis, patients with a history of atrial fibrillation or atrial flutter, with pacemakers and/or ICDs) and those lacking an ECG made within the 3 months prior to surgery were excluded. Additionally, ECGs deemed to be of poor quality were also excluded.

### Perioperative Work-up and Anesthesia

Preoperatively, patients receive a routine medical work up including a 12-lead ECG, a transthoracic echocardiogram, and a coronary angiogram. Additionally, a number of specific examinations for cardiac surgery were performed at the surgeon’s discretion.

As customary at our institution, patients generally did not receive a premedication. Neither B-blockers nor amiodarone were administered *de novo* but were continued. Anesthesia was generally induced with fentanyl (1–4 μg/kg), propofol (1–2 mg/kg), and rocuronium (0.8 mg/kg) and maintained with sevoflurane titrated to achieve a Bispectral Index (BIS) value between 40 and 60. On-pump cardiac surgery is performed either with extracorporeal circulation or minimal extracorporeal circulation (for isolated Coronary artery bypass graft [CABG] surgery) using St. Thomas cardioplegia or blood cardioplegia.

At the end of the surgery, temporary epicardial pacing wires were placed on the surface of the right atrium and ventricle, and pacing leads were connected to a standard external dual-chamber pacemaker (Osypka Pace 203H). Before beginning the weaning process, the pacemaker’s sensing and pacing thresholds for the atrium and ventricle were tested, and separation commenced with a targeted heart rate of 90/min paced either A00 or—if not possible—by DVI pacing modes. Upon arrival in the ICU, patients were first weaned from catecholamines (if administered) and then the pacemaker was weaned according to hemodynamic stability.

### ECG-Based Measurements

**Figure 1.**
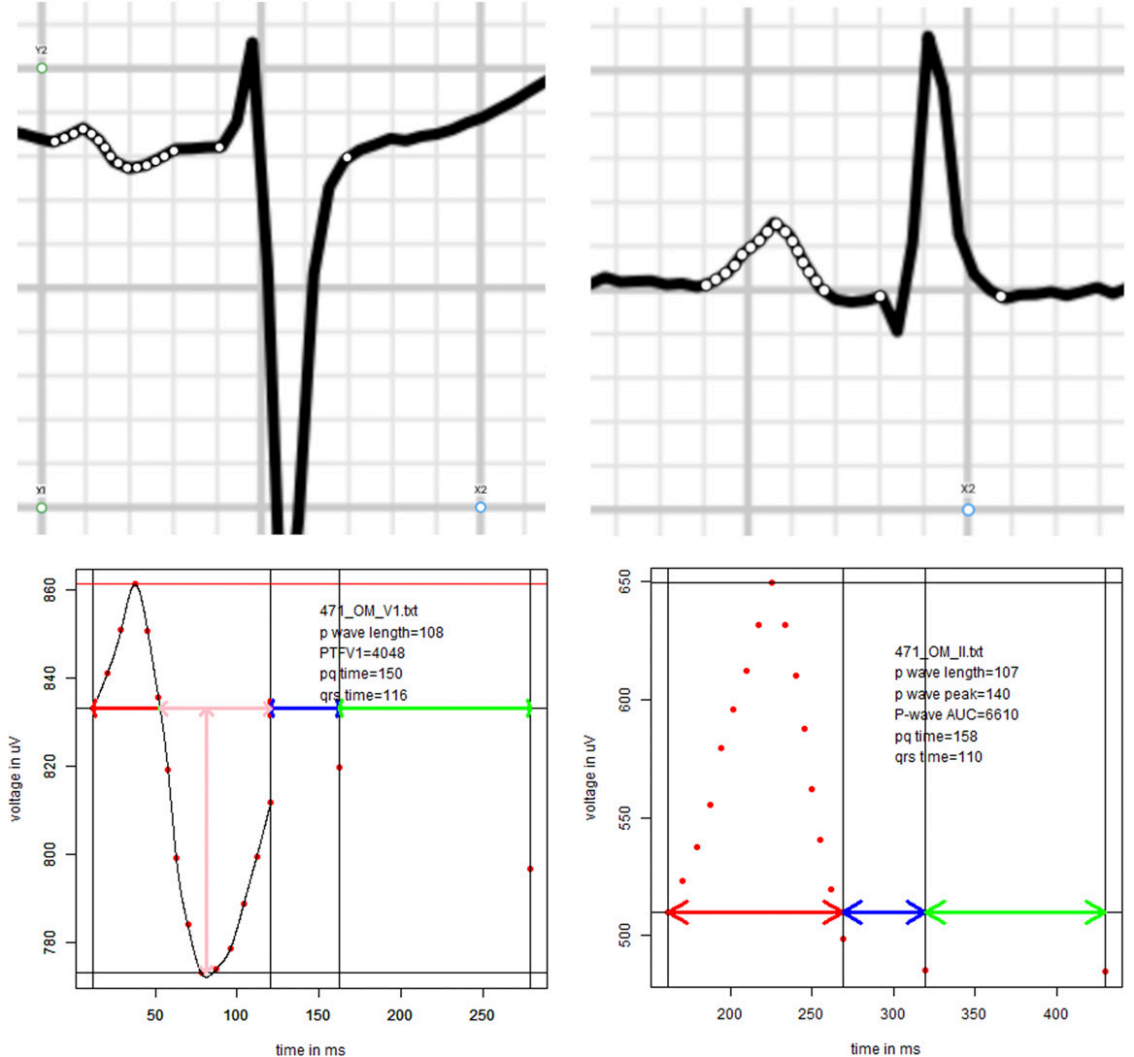
Methodology for ECG-based measurements. Shown are the leads V1 and II (left and right, respectively). The top panels show a scan of the ECG region of interest and the points placed to track and export the p-wave and relevant points. The bottom panels show the measured values using customized R code.

Two authors (M.G. and J.K.B.) analyzed the patients ECGs without knowing whether they later developed atriall fibrillation after the surgery evaluated 252 preoperative 12-lead ECGs (speed 25 mm/s). ECGs were recorded at most 3 months prior to surgery and stored in the patients’ electronic record. In order to most accurately quantify P-wave related measurements, ECGs were first digitally scanned in PNG format and then enlarged (1200%) to show the area of interest. In the next step, edited photos of P-waves in ECGs were examined by Plot Digitizer (Version 1.4.1, D. Medeiros, Figure 1 top), a software which allows placement and exportation of x and y coordinates on images. These coordinates were exported and analyzed by a custom-made code in R (The R Foundation for Statistical Computing, Vienna, Figure 1 bottom).

This process allowed for a detailed and uniform analysis of relevant measurements. Specifically, we examined the PTFV1 (the area under the baseline in the second portion of the biphasic P in V1, as previously described,^17^ P-wave length in lead II, the area under the P-wave in lead II, PR-interval, and QRS duration in lead V1 and II.

### Data Collection, End Points, and Bias

Variables were extracted in the original study from the electronic patient record as was the occurrence of diagnosed new-onset POAF. To accurately describe the patient population, the following pre- and perioperative variables were predefined: age, gender, heart failure, hypertension, diabetes mellitus, stroke, TIA (transient ischemic attack), thrombosis, vascular disease, CHA_2_DS_2_VASC-Score, body mass index (BMI), COPD, obstructive sleep apnea syndrome (OSAS), creatinine clearance, renal failure (defined as glomerular filtration rate [GFR] less than 50 mL/min), mitral valve disease, valve surgery, potassium levels upon ICU, LAVI (left atrial volume index in cm^2^/kg), and left atrial (LA) dilation.

Due to the high correlation between the CHA2DS2VASC-Score and other significant variables (such as age), the score was not included in the adjusted model to avoid multicollinearity.

### Statistical Analyses

Patient characteristics are presented as mean (standard deviation), median (Q1-Q3), and count (%), for normally distributed continuous data, for non-normally distributed data, and for categorical data, respectively. The distribution was visually assessed by histogram. Potential differences in the distribution of these variables in patients with and without POAF were assessed by *t* test, Wilcoxon tests, and Chi-square test.

An initial unadjusted logistic regression analysis was conducted with the variables shown in Table 2 (e.g., age, CHA_2_DS_2_VASC-Score, and PTFV1). For variables with statistically significant odds ratios, a second round of analysis using adjusted logistic regression models was performed to control for potential confounding variables. In cases of strong collinearity, the variable with the strongest association was selected.

The sample size was determined by the following estimation: Applying a rule of thumb that 20 events are required per variable in multivariable models, a total of 60 events would allow for a robust univariable analysis of the PTFV1 as well as for its incremental value in addition to the CHA_2_DS_2_VASC-Score and one other variable. The rule of thumb, as a simplified principle in statistics, refers in this case to determining the number of events needed to ensure adequate power in our analysis. Assuming a POAF incidence of 25% in our population, a total of 240 patients would have to be included in our analysis to arrive at 60 events. Allotting for 5% missing patient characteristics, 4 patients (or 1 POAF) were added per estimator, yielding 252 patients.

All analyses were conducted in R 3.5.2 (The R Foundation for Statistical Computing, Vienna).

## Results

**Figure 2.**
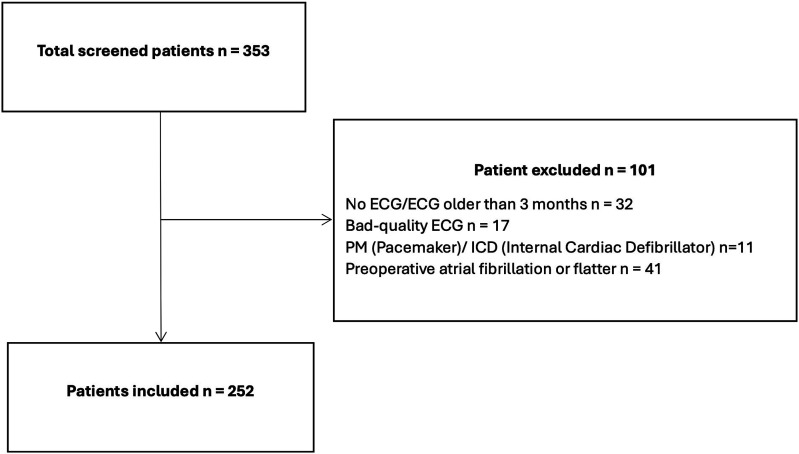
Flow chart of included and excluded patients.

Figure 2 shows a flow chart of patient inclusion. We screened 353 patients underlying on-pump cardiac surgeries, of which 101 were excluded (32 missing ECGs, 17 ECGs with insufficient quality, 11 pacemakers or ICDs, and 41 patients with a history of atrial fibrillation). This left 252 patients for analyses of whom a total of 62 patients (24.6%) developed new-onset POAF during their hospital stay.

A summary of patient characteristics and perioperative information for patients developing and not developing POAF is shown in Table 1. Patients developing POAF tended to be older, exhibited a higher CHA_2_DS_2_VASC-Score, and had poorer renal function. When reported, ECG assessment of the left atrium showed larger atria to be associated with POAF. In terms of surgical factors, patients developing POAF were more likely to receive more than one surgical procedure and exhibited lower postoperative hemoglobin. Patients with POAF had a greater ICU and hospital length of stay.

**Table 1.** Comparison of Patient Characteristics by POAF Status. Patient Characteristics are Presented as Standard Deviation, Median (Q1–Q3), and Count (%). Unless Otherwise Stated Data was Available for all 241 Patients.

Characteristic/Variable	POAF (n = 62)	No POAF (n = 179)	*P*-value
Patient data			
Age	73 [65 to 76]	65 [58 to 73]	<0.001
Sex	18 (29%)	32 (18%)	0.092
BMI	27 [24 to 30]	28 [25 to 30]	0.332
Hypertension	46 (74%)	126 (70%)	0.684
Heart failure history	20 (32%)	52 (29%)	0.753
OSAS	5 (8%)	14 (8%)	1
COPD	8 (13%)	29 (16%)	0.677
Stroke/TIA	7 (11%)	19 (11%)	1
Diabetes	14 (23%)	58 (32%)	0.195
Vascular disease	48 (77%)	144 (80%)	0.743
CHA_2_DS_2_VASC	4 [3 to 5]	3 [2 to 4]	0.024
Creatinine clearance	76 [64 to 87]	92 [72 to 115]	0.001
Mitral disease	15 (24%**)**	25 (14%)	0.095
LVEF	41 (66%)	118 (66%)	0.811
>55%	9 (15%)	33 (18%)	
45–54%	6 (10%)	14 (8%)	
35–44%	5 (8%)	9 (5%)	
25–34%	1 (2%)	5 (3%)	
<25%			
Preoperative β-Blocker	28 (45%)	81 (45%)	1
LAVI(n = 173)	40 [29 to 50]	32 [25 to 38]	0.001
LA dilated(n = 205)	31 (61%)	58 (38%)	0.006
Surgical data			
Elective surgery	49 (79%)	148 (83%)	0.653
Weight of surgery	26 (42%)	106 (59%)	0.023
Isolated CABG	15 (24%)	42 (23%)	
Single non-CABG	19 (31%)	30/179 or 17%	
2 procedures	2 (3%)	1 (1%)	
3 procedures			
ECC time	112 [97 to 142]	107 [90 to 129]	0.148
Postoperative data			
Potassium on ICU arrival (n = 238)	4.5 [4.1 to 4.7]	4.4 [4.1 to 4.7]	0.762
Postoperative hemoglobin	9.0 [8.1 to 10.0]	9.4 [8.4 to 10.7]	0.040
Blood in drains 24 h (n = 226)	810 [420 to 1125]	710 [500 to 900]	0.145
ICU Length of stay	48 [23 to 117]	24 [21 to 49]	0.001
Hospital length of stay	9.8 [7.1 to 13.2]	7.9 [6.8 to 10.6]	0.018

BMI: body mass index, CABG: coronary bypass graft, COPD: chronic obstructive pulmonary disease, ECC: extracorporeal circulation, LA: left atrium, LAVI: left atrium volume index, LVEF: left ventricular ejection fraction, OSAS: obstructive sleep apnea syndrome, POAF: postoperative atrial fibrillation.

Table 2 shows the odds ratios for POAF. The CHA_2_DS_2_VASC-Score was associated with higher odds of POAF (OR 1.242 [1.024 to 1.517]), although this was driven by age (OR 1.079 [1.042 to 1.121]). Additionally, creatinine clearance, valve surgery, and an increasing LAVI exhibited ORs of 0.983 [0.972 to 0.993], 2.43 [1.353 to 4.413], and 1.039 [1.014 to 1.066], respectively. None of the ECG-based measurements were associated with POAF. In the multivariable analysis, only age remained associated with POAF.

**Table 2.** Unadjusted and Adjusted Odds Ratios (ORs) for Postoperative Atrial Fibrillation (POAF).

	Unadjusted OR	Adj OR, continuous. AIC 183.7
Heart failure	1.163 [0.616 to 2.150]	
Hypertension	1.209 [0.639 to 2.375]	
Age, per year	1.079 [1.042 to 1.121]	1.048 [1.001 to 1.102]
Age, 65–75	1.530 [0.844 to 2.759]	
Age, >75	2.463 [1.258 to 4.779]	
Diabetes	0.608 [0.302 to 1.168]	
Stroke, TIA; thrombosis	1.072 [0.401 to 2.585]	
Vascular disease	0.833 [0.420 to 1.720]	
Female sex	0.532 [0.274 to 1.051]	
CHA_2_DS_2_VASC score, per point	1.242 [1.024 to 1.517]	
COPD	0.766 [0.311 to 1.710]	
OSAS	1.034 [0.323 to 2.837]	
Creatinine clearance	0.983 [0.972 to 0.993]	0.991 [0.976 to 1.005]
Mitral valve disease	1.966 [0.943 to 4.003]	
Valve surgery, any	2.43 [1.353 to 4.413]	1.933 [0.882 to 4.269]
Potassium upon arrival on the ICU	0.991 [0.543 to 1.773]	
LAVI, per cm^2^/kg	1.039 [1.014 to 1.066]	1.025 [0.998 to 1.054]
Dilated LA	2.566 [1.349 to 4.974]	
PTFV1, per 100 ms*uV	1.004 [0.997 to 1.010]	
PTFV1 <4000 ms*uV	0.967 [0.542 to 1.726]	
P-wave length in lead II	0.992 [0.978 to 1.005]	
P-wave length>110 ms	1.233 [0.570 to 2.906]	
P-wave AUC in lead II	1.000 [0.999 to 1.001]	
PR-interval	1.004 [0.996 to 1.012]	
QRS duration	1.001 [0.989 to 1.016]	

AIC: akaike information criterion, AUC: area under the curve, COPD: chronic obstructive pulmonary disease, ICU: intensive care unit, LA: left atrium, LAVI: left atrium volume index, OSAS: obstructive sleep apnea syndrome, OR: odds ratio, POAF: postoperative atrial fibrillation.

## Discussion

In this observational retrospective analysis, we found no association between PTFV1 or other ECG-based variables and new onset POAF in patients undergoing cardiac surgery with cardiopulmonary bypass. However, age, creatinine clearance, valve surgery, and LAVI were associated with a higher incidence of POAF with age seeming to be the most robust.

### Comparison to Previous Studies Examining PTFV1

To the best of our knowledge, this is the first study to investigate PTFV1 in the postoperative cardiac surgery population. The PTFV1 was first described by Morris et al.^16^ dividing the P-wave in lead V1 into initial and terminal portions. They were able to show that normal subjects could be distinguished from patients with left-sided valvular lesions.

For non-surgical scenarios, a meta-analysis comprising 12 studies up to August 2018 and involving over 50,000 patients highlighted that an abnormal PTFV1 >0.04 mm*s was associated with atrial fibrillation (pooled OR: 1.39 ;95% CI 1.08 to 1.79). When viewed as a continuous variable, a pooled OR per 1 standard deviation change was 1.27 (95% CI 1.02–1.59) as documented by Huang et al.^15^ Interestingly, the subgroup analysis revealed that this association was absent in European populations, who were also generally younger (OR 1.05, 95% CI 0.91 to 1.20). Other broad cohort studies have supported an association in the general non-surgical population,^14,18,19^ as well as in specialized non-surgical populations (ischemic stroke, left atrial overload, myocardial infarction, and patients with renal failure).^15^ However, it is worth noting that there are disparities among these individual studies. These variations can be attributed to the multifaceted nature of PTFV1, which encompasses factors such as left atrial (LA) enlargement, increased LA hypertrophy, elevated LA pressure, and aberrant interatrial conduction.^20^ Interestingly, Kanzaki et al.^20^ also showed that PTFV1 might be a valuable predictor for the reoccurrence of atrial fibrillation in patients undergoing cryoballoon ablation (Table 3).

**Table 3.** An Overview of Studies That Explore AF in Relation to PTFV1.

	Patients	Analysis	Comparisons	Outcome	Main Results
^13^	N = 10,647, male: 5622	PTF, HR, PR interval, QRS duration, QTc interval, LVH	• Prevalence and prognostic significance of an abnormal PTF in general population	PTF	• Subjects with abnormal PTF: older, higher BMI, and higher BP
	Age: mean 44	Lead: standard 12-ECG			• Increased PTF with CVD, cardiac medication, LVH, MI
	Finnish population				• PTF >0.06 mmXs associated with a higher risk for AF
					• PTF represents structural and functional changes in left atria
^21^	N = 226, male: 134	PTFV1, HR, QTc, LA, clinical factors	• With and without AF	AF	• 16 (7.1%) with AF
	Age: mean 84	Lead: standard 12-ECG	• Compared clinical parameters		• Increase in PTFV1 per 0.01 mmXs and age strongly associated with AF
	Ischemic stroke patients				
^14^	N = 51,372	Meta-analysis of 11 studies	• PTFV1 abnormal defined as > 0.04 mm/s	AF	• As categorical variable increased risk for AF by 39% vs continuous 27%
	Non-surgical patients in studies		• Comparison in subgroups general population, acute ischemic stroke, and hemodialysis		• Weaker association depending where (country) conducted
					• Three subgroups general population, stroke, and hemodialysis
^20^	N = 78, male: 52	p-wave area, PD, amplitude of initial and terminal portion of the p-wave	• With and without AF	AF	• 15 patients (19%) developed AF
	Age: mean 55.8	Lead: standard 12-ECG, V1	• Two subgroups ≥65 years and <65 years		• P-wave initial portion in V1 independent risk factor for AF
	Non-surgical with LA overload		• Overload defined as PTF >0.04s and >0.1 mV		• Overload LA/RA leads to remodeling and interferes with conduction
				
^20^	N = 79, male: 56	PD, FPD, PTFV1	• Recurrence AF yes/no	AF	• Recurrence in 11 patients (14%)
	Age: mean 63.4	Lead: standard 12-ECG V1, SAECG p-wave duration			• PTFV1 calculated from amplitude in V1 times FPD in SAECG more accurate and independent predictor of AF recurrence
	Paroxysmal AF patients after pulmonary vein isolation				
^17^	N = 101, male: 81	PTFV1, atrial function with CMR and CaMKII activity	• MI patients with kidney donors as control cohort	Atrial function	• PTFV1 as independent marker for early atrial cardiomyopathy characterized by electrical remodelling
	Age: mean 57.9	Lead: standard 12-ECG	• CABG patients used for biopsies		• Abnormal PTFV1 predicting atrial pro-arrhythmic activity
	56 MI patients, 13 kidney donors, 32 CABG patients		• Normal and abnormal PTFV1		
				
^17^	N = 5667, male: 2577	PD, PTF, P-wave axis	• Baseline and follow-up population compared p-wave indices with different cut offs	Abnormal p-wave, AF	• Prevalence and incidence rates of p-wave abnormalities are relatively high in the general population
	Age: mean 51.5	Lead: standard 12-ECG	• PD ≤120 ms and >120 ms		• PTF: <6mVms association with AF but not when <4mVms
	Finnish population				• Only prolonged PD predicted AF in follow-up
^18^	N = 11’364, male: 4860	PR interval, maximum PD, maximum p-wave area, PTF	• Two study groups FHS (multigenerational) and ARIC (biracial) developing AF	AF	• P-wave indices add novel information about atrial electrical function but have highly limited contribution toward enhancing AF risk modeling metrics
	Age: mean 62.3	Lead: standard 12-ECG			
	American population				
^22^	N = 262, male: 149	AV-block, right atrial enlargement, PTF, premature atrial contraction, branch block, axis deviation	• With and without AF	AF	• Prevalence depending on age groups, 4.2%, 14.2% and 20.3%, respectively
	Age: mean 62.2	Lead: standard 12-ECG, V1 and V5/6	• Compared in three subgroups divided by age		• PTF independently associated with new onset AF
	Hemodialysis patients		• PTF defined >0.04 mm/s		
^23^	N = 166, male: 97	P-wave dispersion, Pi, IAB	• Endstage renal disease with control group	Pi, IAB	• Pi was significantly higher in HD
	Age: mean 53	Lead: standard 12-ECG			• Prevalence of IAB in both HD and CAPD subgroups
	Patients with end-stage renal disease				

AF: atrial fibrillation, BP: blood pressure, CABG: coronary artery bypass graft, CAPD: continuous ambulatory peritoneal dialysis, CMR: cardiac magnetic resonance imaging, CVD: cardio vascular disease, FPD: filtered P-wave duration, HD: hemodialysis subgroup, HR: heart rate, IAB: interatrial block, LA: left atrium, LVH: left ventricular hypertrophy, MI: myocardial infarction, PD: p-wave duration, Pi: p-wave index, PTFV1: p-wave terminal force in lead V1, RA: right atrium, SAECG: p-wave signal-averaged electrocardiogram.

### Comparison to Previous Studies Examining ECG Based Markers and POAF in Cardiac Surgery

Table 4 summarizes studies examining ECG-based predictors of POAF in patients undergoing CABG, valve surgery, and combined procedures. However, none of these studies examined PTFV1. The most commonly examined ECG-based predictor was P-wave duration and P-wave dispersion. While the majority of published studies examining P-wave duration found an association with POAF, P-wave dispersion was less clear. Of note are the three larger studies. Rader et al. examined over 13,000 patients undergoing a range of cardiac surgeries, of which 58% underwent isolated CABG.^25^ They found that P-wave duration, amplitude, as well as the PR interval and QRS duration, improved the prediction of POAF over clinical predictors (such as older age, higher body mass index, history of atrial arrhythmia, lager left atrial volume and preoperative beta-blocker use) alone. Furthermore, they differentiated between Caucasian and African American populations (lower risk in African American patients); patients with pacemakers – but not those with a history of atrial fibrillation – were excluded from the study. Similarly, Amar et al. found P-wave duration to be weakly associated with POAF in some 1500 patients undergoing CABG surgery without concurrent valvular surgery.^35^ Additionally, these patients had no history of prior cardiac surgery but did have a history of atrial fibrillation. The study population was almost equally divided in terms of gender but was generally older with a mean age of 68 years. Finally, Wong et al. examined premature atrial contraction, P-wave index, P-frontal axis, P-wave dispersion, and the PR interval in some 500 patients undergoing CABG and valve surgeries.^27^ Interestingly, 70% of patients who had valve surgery developed POAF, as compared to only 30% in the CABG group. Participants were excluded if their preoperative ECG did not show a sinus rhythm or if they had atrial pacing, but a history of AF was not an exclusion criterion. The mean age was 70.3 years, making it older on average than the population studied by Amar et al.^35^ Additionally, Wong et al. took into account the racial backgrounds of participants, including Asian, Black, White, and Hispanic.^27^ However, the univariate analysis did not show a significant association between POAF and race. They found premature atrial contraction, the P-wave index, and the p-frontal axis to be independently associated with POAF, while the other ECG-based measurements were not. A few reasons may explain these divergent results, such as different and/or unspecified cut-offs for measurements,^26,31,35^ different filtering techniques,^30^ the inclusion of patients with differing demographic backgrounds (gender, race) and patient characteristics (e.g., history of atrial fibrillation). Furthermore, several factors such as myocardial fibrosis, vagal tone, and certain medications, can influence conduction in the atria or AV node.^37^ The multifactorial etiologies of POAF have led some authors to conclude that it ultimately remains uncertain whether measurements from a standard 12-lead ECG can predict POAF.^35^

**Table 4.** An Overview of Studies That Explore AF in Relation to PTFV1 With Focus on POAF.

	Patients	Intervention	Comparisons	Outcome	Main results	Appeared in
^24^	N = 42, male: 34	P-wave dispersion,	• With and without POAF	POAF	• POAF 2 day 28.6%	Current Problems in Cardiology
	Age: mean 57 CABG only	LAVI	• No cut-off		• P-wave dispersion longer in POAF	
		Lead: standard 12-ECG			• LAVI no difference	
^25^	N=1,553, male: 1,040	PD	• With and without POAF	POAF	• POAF 33%	Journal of the American College of Cardiology
	Age: mean 68 CABG only	Lead: standard 12-ECG	• PD in general and >110 ms		• PD associated with POAF, a weak but independent risk factor	
					
^26^	Correspondence in reference to 27	PD	With and without POAF	POAF	• Various studies have different cut-offs for PD because of varying filtering techniques applied	The Society of Thoracic Surgeons
		Lead: standard 12 ECG, SAECG				
^28^	N = 101, male: 79	FPD and RMS20, CHRS	• With and without POAF	POAF	• POAF in 37 patients (37%)	International Journal of Cardiology
	Age: mean 68.2 CABG only	Lead: SAECG	• 124 ms as cut-off for PD		• All 3 (FDP, RMS20 and CHRS) predict POAF	
					• Different cut-off points in other studies lead to different outcomes.	
^29^	N = 151, male: 126	pCR, PMD, integral of p-wave, prolonged PD, RMS20	• With and without POAF	POAF	• POAF 26%	Journal of Cardiovascular Electrophysiology
	Age: mean 65 CABG only	Lead: standard 12-ECG and SAECG	• POAF defined as lasting >10 min or need for intervention		• Simple ECG indices identify risk for POAF	
					• PD is a limited predictor or controversial	
					• Pi is significantly greater in Patients with POAF	
^30^	N = 100, male: 69	Five ECG markers: (QRS duration, PR interval, PD, LAE, LVH	With and without POAF (50/50)	POAF	• POAF 50% (matched)	Journal of Cardiothoracic and Vascular Anesthesia
	Age: mean 69.6 CABG, valve replacement, combined				• Combined approach clinical signs and ECG, respectively age, PR interval, QRS duration and LAE	
		Clinical signs (age, gender, BMI, type of surgery)			• Adding ECG markers to clinical signs improved AUROC from 0.54 to 0.67	
		Lead: QRS/PR-interval any, LAE II or V1			• First ones to use combined approach	
^31^	N = 52, male: 40	P-wave dispersion, mean PD, EF	• With and without POAF	POAF	• 8 patients (15.38%) developed POAF	Atherosclerosis Journal
	Age: mean 57.82 CABG	Lead: standard 12-ECG	• Patients older than 70 years were excluded		• P-wave dispersion and mean PD are the main independent predictors for POAF	
					
^11^	N = 30, male: 23	P-wave dispersion and duration, DMI	With and without POAF	POAF	• P-wave dispersion postoperative longest in those with POAF	Journal of Cardiothoracic Surgery
	Age: mean 62 CABG	Lead: standard 12-ECG			• DMI equally effective to p-wave dispersion in detecting POAF	
					
^27^	N = 95, male: 60	PD, LA	With and without POAF	POAF	• 28 patients (29%) developed POAF	Ann Thorac Surg
	Age: mean 71.8 CABG, aortic valve replacement	Lead: SAECG			• PD in POAF patients significantly prolonged	
					• LA dimension accurate predictor of the development of POAF	
					
^32^	N = 150, male: 96	RR, PR and QT intervals, linear/non-linear HR	with and without POAF	POAF	• 31 patients (21%) developed POAF	International Journal of Cardiology
	Age: mean 74.3 CABG, aortic valve replacement or combined	Lead: high-resolution ECG			• PR interval non-linear HR and age strongest preoperative predictors for POAF	
					Shorter PR interval and altered cardiac autonomic regulation mirror the concurrent activation of both PNS and SNS preceding the onset of POAF	
^33^	N = 13,356, male: 9513	PD and p-wave amplitude, PR interval, QRS duration, indices of LVH	With clinical predictors only clinical predictors and semi-quantitative ECG diagnosis of atrial enlargement	POAF	4724 (35%) POAF	Journal of Electrocardiology
	Age: mean 64 CABG (58%), valve surgery, combined	Lead: standard 12-ECG			p-wave amplitude in V1 and aVR powerful predictor for POAF	
					Improvement (7%) of the prediction by combining quantitative ECG predictors and clinical predictors	
^34^	N= 36 men: 32	Maximum PD, p-wave dispersion	Two protocols: subgroup with elderly (>60 years) ECG, before and after OHS all patients on the day of OHS as well as after	POAF	p-max significantly decreased immediately after OHS, with no change in p-wave dispersion	Heart & Lung
	Age: mean 57	LEAD: standard 12-ECG, excluding lead III			p-wave dispersion significantly increased on post-OHS day 2 and 3	
	CABG, valve surgery				p-max greatest on day 3	
					
^6^	N = 276, men: 207	Short- and long-term morbidity and mortality	Patients after cardiac surgery with POAF compared to control group (nSR)	POAF	POAF at the time of hospital discharge is a significant predictor of late all-cause mortality, cardiac mortality and permanent/chronic POAF	European Journal of Cardio-Thoracic Surgery
	Age: mean 70.2 CABG		Follow-up			
					
^35^	N = 526 men: 353	PAC,Pi, p-frontal axis, p-wave dispersion, PR interval	Characteristics between patients with and without POAF	POAF	• 208 (39.5%) POAF	Journal of Cardiothoracic and Vascular Anesthesia
	Age: mean 70.3 CABG mitral valve surgery, combined	Lead: SAECG			• PAC, Pi and p-frontal axis independently associated with POAF	
					• Incorporate those indices with prediction models (besides clinical predictors) for POAF	
					
					
^36^	N = 12 studies	Meta-analysis of 12 studies	p-wave indices on standard ECG and SAECG	POAF	• 6/8 demonstrated PD increased risk for POAF, whereas 2/8 showed no difference	International Journal of Cardiology
	CABG				• 3 studies provide p-wave dispersion as predictor for POAF ? Zhang didn’t find association between p-wave dispersion and increased risk for POAF	
					• 7 studies observed strong associations between signal averaged p-waves and POAF	

CABG: coronary artery bypass graft, CHRS: chemoreflexsensitivity, DM: doppler myocardial imaging, EF: ejection fraction, FPD: filtered p-wave duration, HR: heart rate, LAE: LA enlargement, LAVI: left atrial volume index, LVH: left ventricle hypertrophy, nSR: normocardic sinus rhythm, OHS: open heart surgery, PAC: premature atrial contraction, pCR: p-wave complexity ratio, PD: p-wave duration, Pi: p-wave index, PMD: p-wave morphology dispersion, POAF: postoperative atrial fibrillation, RMS20: root mean square voltage, SAECG: p-wave signal-averaged electrocardiogram.

### Potential Mechanisms of New-Onset Postoperative Atrial Fibrillation after (Cardiac) Surgery

Although an abnormal PTFV1 may be promising in predicting atrial fibrillation in a non-surgical population,^15^ it may be less relevant in cardiac surgery. This lack of association may be due to the different mechanisms of new-onset postoperative atrial fibrillation in cardiac surgery patients.^38^

The type of surgical procedure and the surgical steps themselves may play a distinctive role. Patients undergoing valve surgery have a higher incidence of early postoperative AF than patients after CABG.^39^ Moreover, off-pump CABG is associated with a lower incidence of AF than traditional on-pump CABG.^40^ A recently conducted large randomized trial examined the effect of a left posterior pericardiotomy.^41^ A total of 420 patients undergoing elective cardiac surgery (excluding patients scheduled for mitral or tricuspidal valve surgery) were included in the study. POAF occurred in 37 (17%) of 212 patients having left posterior pericardiotomy compared to 66 (32%) of patients with no intervention. The large effect size of this surgical step not only highlights the relevance of surgery and surgical steps in POAF, it also describes a potential pathomechanism in which blood and clots accumulating in the pericardial cavity trigger POAF. However, whether pleural effusion induces the pathomechanism of an inflammatory response or the exertion of compression and stretching of the atrial wall is not well understood.^21^

If, as suggested, the negative terminal part of the P-wave serves as an indicator of heightened atrial fibrosis, electrical remodeling, and structural changes in the atrium, the effect in surgical patients may simply be relatively smaller due to the impact of the surgical procedure.

### Clinical Implications

Current guidelines on POAF recommend perioperative oral B Blocker and perioperative amiodarone for the prevention of postoperative AF (Class I, A/B, Class IIa A/B).^8^ Additionally, pre, intra, and postoperative prophylactic amiodarone are reasonable in elevated-risk patients. However, identification of elevated-risk patients remains difficult with no specific recommendation made (risk factors: age, history of atrial fibrillation, renal failure, mitral valve surgery/disease, heart failure, COPD; no cut-off for elevated risk made). It seems ECG-based measures may be of limited value. Of the available scores, the CHA_2_DS_2_VASC and POAF Score have been recommended to stratify risk. While our study showed the CHA_2_DS_2_VASC-Score to be associated with POAF, it was largely driven by age and without model improvement for the CHA_2_DS_2_VASC-Score (age AIC = 257.1, CHA_2_DS_2_VASC AIC = 271.1). The POAF score incorporates a number of variables not applicable to our patients (preoperative IABP, emergency surgery) and was hence not calculated. However, it is largely based on age and in addition to the above, it includes COPD, GFR, heart failure, and valve surgery, all of which were included in our sample.

### Study Strengths and Limitations

This study had a number of strengths. First, it is one of the larger studies examining P-wave signs for POAF after cardiac surgery and the only one to date examining PTFV1 in a cardiac surgery population. Second, we included CABG and non-CABG patients, which may have increased heterogeneity in the sample but led to larger external validity. Third, several different P-wave signs were analyzed in a detailed fashion.

However, our study also has some limitations. First, it was a single center, retrospective secondary analysis, and—as such—deserves all the inherent caveats. Second and relatedly, as a secondary analysis examining native P-waves in patients with no history of atrial fibrillation, some patients were excluded (e.g., paroxysmal atrial fibrillation and pacemakers). These patients still require management and may exhibit the highest risk. Third, preoperative 12-lead ECGs were examined and hence no conclusions can be drawn about intraoperative or postoperative ECGs.

## Conclusion

Our study found no association between PTFV1 and the incidence of POAF after cardiac surgery. Clinical characteristics (e.g., age and renal function) and surgical considerations (type of surgery) seem to be more important in the risk assessment for new-onset POAF after cardiac surgery. For non-cardiac surgery, PTFV1 remains uncertain as there is no interaction on the heart itself.

There remains an urgent need for standard care protocols for cardiac surgery patients, given the consistent or even rising incidence of POAF and the potential adverse effects of atrial fibrillation and antiarrhythmic drugs used for thromboembolism prophylaxis in atrial fibrillation patients.^42^
